# Psychosocial factors affecting COVID-19 vaccine uptake in the UK: A prospective cohort study (CoVAccS – Wave 3)

**DOI:** 10.1016/j.jvacx.2023.100276

**Published:** 2023-02-13

**Authors:** Louise E. Smith, Julius Sim, Megan Cutts, Hannah Dasch, Richard Amlôt, Nick Sevdalis, G. James Rubin, Susan M. Sherman

**Affiliations:** a Institute of Psychiatry, Psychology and Neuroscience, King’s College London, UK; b NIHR Health Protection Research Unit in Emergency Preparedness and Response, UK; c School of Medicine, Keele University, UK; d School of Psychology, Keele University, UK; e Centre for Implementation Science, King’s College London, UK; f Behavioural Science and Insights Unit, UK Health Security Agency, UK

**Keywords:** COVID-19, Vaccination, Intention, Uptake, Behaviour, Refusal

## Abstract

**Background:**

We investigated factors associated with COVID-19 vaccine uptake, future vaccination intentions, and changes in beliefs and attitudes over time.

**Methods:**

Prospective cohort study. 1500 participants completed an online survey in January 2021 (T1, start of vaccine rollout in the UK), of whom 1148 (response rate 76.5 %) completed another survey in October 2021 (T2, all UK adults offered two vaccine doses). Binary logistic regression analysis was used to investigate factors associated with subsequent vaccine uptake. Content analysis was used to investigate the main reasons behind future vaccine intentions (T2). Changes in beliefs and attitudes were investigated using analysis of variance.

**Findings:**

At T2, 90.0 % (95 % CI 88.2–91.7 %) of participants had received two doses of a COVID-19 vaccine, 2.2 % (95 % CI 1.3–3.0 %) had received one dose, and 7.4 % (95 % CI 5.9–8.9 %) had not been vaccinated. Uptake was associated with higher intention to be vaccinated at T1, greater perceived vaccination social norms, necessity of vaccination, and perceived safety of the vaccine. People who had initiated vaccination reported being likely to complete it, while those who had not yet received a vaccine reported being unlikely to be vaccinated in the future. At T2, participants perceived greater susceptibility to, but lower severity of, COVID-19 (*p* < 0.001) than at T1. Perceived safety and adequacy of vaccine information were higher (*p* < 0.001).

**Interpretation:**

Targeting modifiable beliefs about the safety and effectiveness of vaccination may increase uptake.

## Highlights

• Prospective cohort study investigating COVID-19 vaccination.
• More people were vaccinated than previously reported intending to be vaccinated.
• People who had received at least one vaccine were likely to complete the schedule.
• Those who were not vaccinated reported being unlikely to receive a vaccine.
• Uptake was associated with higher intention, social norms, necessity and safety.

## Introduction

One of the main lines of defence against COVID-19 has been vaccination. In the United Kingdom (UK), intention to receive a COVID-19 vaccine when one became available was reasonably high, with 74 % indicating that they were likely to be vaccinated against COVID-19 in a survey conducted by our team in January 2021 (at the start of the vaccine rollout) [1]. Other UK studies have found comparable rates of intention to be vaccinated (63 % to 89 %) [2], [3], [4]. Differing rates can be explained by different timepoints in the pandemic and different questions used. On 19 July 2021, all UK adults had been offered a first dose of a COVID-19 vaccine [5]. At this point, people were eligible for their second vaccine eight weeks after they had received their first, meaning that all UK adults would have been offered a full course by 13 September 2021. Box 1 shows a timeline of pertinent dates relating to the vaccine rollout in the UK.

Box 1 **. Timeline of dates relating to the COVID-19 vaccine rollout for adults in England.**

**Table:** 

8 December 2020. Vaccine offered to residents in care homes for older adults and their carers, and those aged 80 years and over [6]. First COVID-19 vaccine administered in UK [7]. 31 December 2020. Vaccine offered to frontline health and social care workers [8]. 18 January 2021. Vaccine offered to those aged 70 years and over, and clinically extremely vulnerable adults [9]. 15 February 2021. Vaccine offered to those aged 65 years and over, and those aged 16 to 64 years with underlying health conditions [10]. 1 March 2021. Vaccine offered to those aged 60 years and over [11]. 8 March 2021. Vaccine offered to those aged 56 years and over [12]. 17 March 2021. Vaccine offered to those aged 50 years and over [13]. 7 April 2021. Possible link between AstraZeneca vaccine and very rare cases of unusual blood clots with low blood platelets [14]. Joint Committee on Vaccination and Immunisation (JCVI) recommend that those aged 30 years and younger are offered an alternative vaccine [15]. 13 April 2021. Vaccine offered to those aged 45 years and over [16]. 26 April 2021. Vaccine offered to those aged 42 years and over [17]. 30 April 2021. Vaccine offered to those aged 40 years and over [18]. 13 May 2021. Vaccine offered to those aged 38 years and over [19]. 18 May 2021. Vaccine offered to those aged 36 years and over [20]. 20 May 2021. Vaccine offered to those aged 34 years and over [21]. 22 May 2021. Vaccine offered to those aged 32 years and over [22]. 26 May 2021. Vaccine offered to those aged 30 years and over [23]. 8 June 2021. Vaccine offered to those aged 25 years and over [24]. 15 June 2021. Vaccine offered to those aged 23 years and over [25] 16 June 2021. Vaccine offered to those aged 21 years and over [26]. 18 June 2021. Vaccine offered to those aged 18 years and over (all adults) [27]. 16 September 2021. First booster vaccines (third dose) offered to residents in care homes for older adults, those aged 50 years and over, frontline health and social care workers, those aged 16 to 49 years with underlying health conditions, and adults household contacts of immunosuppressed individuals who had their second dose at least six months previously [28]. 15 November 2021. Joint Committee on Vaccination and Immunisation (JCVI) recommend that booster (third) doses be offered to all those aged 40 years and over [29]. 12 December 2021. Booster vaccine (third dose) offered to all those aged 30 years and over [30]. 31 December 2021. All adults offered booster vaccine (third dose) [31].

Most studies investigating COVID-19 vaccination uptake have explored factors associated with intention to receive a vaccine using cross-sectional survey methods, finding that vaccination intention is associated with psychological, contextual and sociodemographic factors. In the UK, vaccination intention has been associated with: greater perceived necessity of the vaccine, lower perceived safety concerns, believing that others like you will be vaccinated (i.e. more supportive perceived social norms), and perceiving a low risk of infection [1], [2], [4]. Not intending to be vaccinated has been associated with not having received an influenza vaccine last year and lower adherence to other Government guidelines [1], [4]. Sociodemographic factors associated with not intending to be vaccinated have included: lower income, lower education, belonging to a minoritized ethnic group, younger age, being female, and living with a dependent child [4], [32].

While these studies informed communication campaigns at the start of the vaccine rollout, there are known differences between intended and enacted health behaviours [33]. To the best of our knowledge, there are very few studies investigating psychological and contextual factors (i.e., not sociodemographic factors) associated with COVID-19 vaccine uptake in the UK general population. Among UK healthcare workers, not having had a COVID-19 vaccine was associated with previous confirmed SARS-CoV-2 infection, as well as younger age, being female, greater deprivation, and belonging to a minoritized ethnic group [34].

Globally, few longitudinal studies exist investigating vaccine uptake. One conducted in China found that previous vaccination intention (before the start of the vaccination campaign) and believing that the vaccine was safe were associated with vaccine uptake, whereas vaccine shortages were associated with not being vaccinated [35]. In a study of students (aged 17 to 28 years) in the Netherlands, vaccination intention (when COVID-19 vaccines were approved but not yet available for young adults) was associated with later uptake [36]. Greater worry (measured before COVID-19 vaccines were approved) was associated with vaccination intention; mediation analyses indicated that there was an indirect effect of greater perceived severity of COVID-19 (measured before COVID-19 vaccines were approved) on uptake, through worry and vaccination intention. In Israel, vaccination intention (measured in the week before a COVID-19 vaccine was made available to the general public) was strongly associated with later behaviour (measured after vaccinations were available for all individuals) [37]. COVID-19 illness and vaccine attitudes and beliefs, perceived social norms, and past influenza vaccination explained 86 % of the variance in vaccination intention, which itself mediated associations with behaviour.

Beliefs about, and attitudes towards, COVID-19 vaccination are likely to have changed over the course of the pandemic, as vaccines were rapidly developed, tested, approved, and rolled out to the population. During the rollout, the AstraZeneca vaccine was linked to unusual blood clots with low blood platelets (published April 2021) [14]. This was the focus of widespread media attention and linked to the suspension of delivery of the vaccine in younger age groups in some countries [38]. Pfizer and Moderna vaccines have also been linked to other very rare adverse effects (myocarditis and pericarditis), although these received less media attention [39]. Research conducted in the United States (US) between March and August 2020 indicated that vaccination intention and general vaccine attitudes became more negative [40]. However, since the start of the rollout, studies indicate more positive vaccine intentions and sentiments, with vaccine refusal and delay decreasing between October 2020 and July 2021 in the US [41]. In Italy, more people agreed that vaccines were important to public health and fewer endorsed the idea that vaccines were created to make money for pharmaceutical companies in May 2021 compared to May 2020 [42]. In a cohort of UK older adults (aged 65 years and over), concerns about commercial profiteering and mistrust of vaccination decreased, while collective responsibility and worries about unforeseen future effects had increased [43].

The aims of this study were to investigate: factors associated with subsequent uptake of a COVID-19 vaccine; changes in beliefs and attitudes about COVID-19, COVID-19 vaccination, and general vaccination beliefs and attitudes between January and October 2021; likelihood of further vaccination (completing or starting vaccine schedule in those partially or not vaccinated, respectively; and likelihood of accepting a booster vaccine); and reasons favouring or disfavouring future vaccination.

## Methods

This study reports data from the second and third rounds of the UK-wide ‘COVID-19 Vaccination Acceptability Study’ (CoVAccS), designated here as T1 and T2. Questions directed to parents about child vaccination are reported elsewhere [62].

### Design

This was a prospective cohort study. Participants completed an online survey at the start of the rollout of the COVID-19 vaccine in the UK (T1, 13–15 January 2021; results published in Sherman et al. [1]) and after the vaccine had been offered to all adults (T2, 4–15 October 2021).

### Participants

Participants were recruited from Prolific’s online research panel. Participants were eligible for the study if they were living in the UK, were aged 18 years or older, and had not completed a previous round of the CoVAccS study (data collected July 2020, designated here as T0) [44]. We recruited 1,500 participants at T1, using quota sampling (based on age, sex, and ethnicity). Full details of data collection at T1 are reported in Sherman et al. [1]. Only participants who had taken part in round 2 of our survey (T1, January 2021) were invited to take part in the third round of data collection (T2, October 2021) and formed the study cohort.

### Measures

Full survey materials are available online [45]. To allow direct longitudinal comparisons, with the exception of demographic questions such as age and gender, the same questions were asked at T1 and T2 [1]. Further questions were added, as detailed below.

### Uptake of vaccine

Participants were asked if they had been vaccinated against coronavirus. Response options were “yes, I’ve had one dose”, “yes, I’ve had two doses”, “no”, “don’t know” and “prefer not to say” (asked at T1 and T2). At T2, participants who reported they had been vaccinated were asked which vaccine they had received (choice of Pfizer-BioNTech, AstraZeneca, Moderna, Janssen [Johnson & Johnson], a made-up brand “Cambriona”, or another vaccine not listed above), to ascertain whether they had completed the full vaccine schedule. They were also asked if they would have preferred a different vaccine from the one they had received, and if so, which vaccine they would have preferred, using the same list.

The following questions were only asked at T2. Participants who reported that they had only had one dose were asked how likely they would be to have a second dose on an 11-point scale from “extremely unlikely” (0) to “extremely likely” (10), and to give the main reason why they were likely or unlikely to have a second dose. These questions were only asked to those who indicated that they had received a vaccine that needed two doses to be “fully vaccinated” (Pfizer-BioNTech, AstraZeneca, Moderna). Participants who had not been vaccinated were asked how likely they would be to get vaccinated using the same 11-point scale, and to give the main reason why they were likely or unlikely to have a vaccine.

We asked participants if they had had a COVID-19 booster vaccination. Those who indicated they had not had a booster were asked how likely they would be to have one if it became available to them.

### Psychological and contextual factors

These questions were asked at both T1 and T2 and were informed by existing psychological theory and evidence on psychosocial factors affecting vaccination uptake [1], [44]. Participants were asked about the perceived risk of COVID-19 to themselves personally, to people in the UK, and to people in their local area (five-point scale from “no risk at all” to “major risk”). We also asked participants if they thought they had had, or currently had, a confirmed COVID-19 infection, and whether they personally knew anyone who had had COVID-19.

We measured participants’ beliefs and attitudes about COVID-19. At T1, eight questions were used, asking about perceived worry about catching COVID-19, perceived susceptibility to and severity of COVID-19, and the impact and management of COVID-19. Questions were answered on an 11-point scale (“strongly disagree” [0] to “strongly agree” [10]).

Perceptions of vaccination were sought at T1 and T2. We measured general vaccine beliefs and attitudes using two items, asking about vaccination in general being a good thing and fear of needles. Beliefs and attitudes about COVID-19 vaccination were elicited using 21 questions, including perceived effectiveness of vaccination, social norms of vaccination, ease of vaccination, novelty and safety of vaccination, and (at T2) whether COVID-19 vaccination should be made mandatory. Questions were phrased to take into account whether the participant had already been vaccinated. All questions were answered using the same 11-point scale (“strongly disagree” [0] to “strongly agree” [10]).

At T1, participants were asked how likely they were to have a COVID-19 vaccination on an 11-point scale from “extremely unlikely” (0) to “extremely likely” (10).

### Personal and clinical characteristics

Participants’ age, gender, ethnicity, religion, education, working situation, household income, and chronic illness status (self and household member if applicable) were collected at T1. As participants could have been diagnosed with a medical condition or changed job roles between rounds of data collection, we asked participants whether they had a chronic illness and about their current working situation at T2. We also asked participants if they had a vaccine for seasonal flu during the winter of 2020/2021.

### Ethics

Keele University’s Research Ethics Committee granted ethical approval for this study (reference: PS-200129). Before beginning the surveys, participants provided informed consent.

### Analysis

#### Uptake of a COVID-19 vaccine at T2

We tabulated the association between categories of vaccination intention at T1 and subsequent vaccination uptake. These categories were designated a priori on the 0–10 scale as follows: 0–2 “very unlikely”, 3–7 “uncertain”, 8–10 “very likely.”[1].

Due to small numbers of participants who were partially vaccinated, we created a binary outcome variable (unvaccinated vs partially/fully vaccinated). We conducted a logistic regression analysis to investigate factors associated with subsequent uptake of COVID-19 vaccination. Explanatory variables were measured at T1, while vaccine uptake (outcome) was measured at T2. For these analyses, we excluded participants who reported that they had already been vaccinated against COVID-19 at T1 (*n* = 30 at T1, *n* = 24 at T2). Explanatory variables were entered into the regression analysis in two blocks, selected *a priori* [1]. In the first block we entered vaccination intention, measured at T1. In the second block we added variables that had been significant predictors of vaccine intention at T1: [1] four principal components representing i) social norms relating to vaccination, ii) perceived necessity of vaccination, iii) perceived safety of the vaccine, and iv) adequacy of information about the vaccine; an item indicating a belief that only those at risk of serious illness should be vaccinated; an item indicating that vaccination was just a way of vaccine manufacturers making money; and receipt of the influenza vaccine last/this winter (completed and intended behaviour combined to give a single binary item). Principal components were derived from analyses of T1 data [1]. The use of blocks allowed us to gauge the predictive strength of vaccination intention both before and after controlling for other potential predictors of vaccination status. The predictive strength of each model was calculated as the Tjur coefficient of discrimination [46]; this statistic can take values between 0 and 1, with higher values indicating greater predictive power. Additionally, the goodness of fit of each model was measured as the deviance and the improvement in goodness of fit in the second model was tested through a likelihood ratio test on the model deviances. As the odds ratios for the predictors in the analysis could not be compared for their magnitude, owing to the different scales on which these variables had been measured, we also calculated standardized coefficients for each predictor [47]. Checks for collinearity were performed and, for the regression analysis, statistical significance was set at *p* ≤ 0.05, with corresponding 95 % confidence intervals (CIs).

### Changes in beliefs and attitudes about COVID-19 illness and vaccination between January and October 2021

In our previous analyses of T1 data, we used principal components analysis to summarize items relating to beliefs and attitudes about COVID-19 (four resulting components from eight items) and COVID-19 vaccination (five resulting components from 21 items; see Sherman et al. [1] for more details). These components were generated to reduce the number of predictors in the regression model. We assessed changes in beliefs and attitudes about COVID-19, COVID-19 vaccination, and general vaccine beliefs and attitudes between T1 (January 2021) and T2 (October 2021) using repeated measures ANOVA. To measure changes in the principal components between T1 and T2, the original component score coefficients from T1 were used to generate corresponding component scores at T2. Effect sizes were calculated as Cohen’s *f*; a value of 0.10 is considered to represent a small effect, a value of 0.25 a medium effect, and a value of 0.40 a large effect [48]. In view of the number of hypothesis tests performed, statistical significance was set at a more stringent *p* ≤ 0.01 for these analyses.

### Future vaccine intentions, and reasons behind intention

Participants’ intention to receive future COVID-19 vaccines – second dose in those partially vaccinated or any COVID-19 vaccine in those not vaccinated, and a booster vaccine (asked to all) – was categorized using *a priori* cut-points (0–2 very unlikely; 3–7 uncertain; 8–10 very likely) [1], [44].

Open-ended answers about participants’ main reasons why they were likely or unlikely to accept future vaccination were analysed qualitatively through content analysis. An emergent coding approach was used, whereby codes were identified from the data [49]. Content analysis was undertaken by two authors (MC and HD), starting with the coding framework generated from analysis of similar data that had been collected at T1 of the CoVAccS study [1]. Statements were jointly coded by these authors; any difference in opinion was resolved through discussion to give a final set of codes. Codes were applied separately to intention to complete the initial vaccine schedule (receive a second dose in those partially vaccinated) and to initiate the COVID-19 vaccine schedule (in those not vaccinated). We report codes by strength of intention to receive future vaccines (very unlikely, uncertain, very likely).

For analyses investigating intentions to receive a booster vaccine, we excluded those who reported already having had a booster (*n* = 25, 2.2 % of sample).

### Role of the funding source

The funding sources had no role in the study design; the collection, analysis, and interpretation of data; writing of the report; or decision to submit the paper for publication.

## Results

### Participant characteristics

Of the 1500 participants who had completed T1, 76.5 % (*n* = 1148) also completed T2. The mean (SD) age of respondents (recorded at T1) was 48.2 (15.1) years and 53.2 % (*n* = 611) were female. The majority (86.1 %, *n* = 988) were of white ethnicity. A higher percentage of participants who completed T2 were female than of those who did not complete T2 (53.2 % versus 44.3 %). Those completing T2 were also older (mean age 48.2 years versus 37.3 years), and more likely to be of white ethnicity (86.1 % versus 80.1 %). The mean vaccination intention score was also higher in those who completed T2 (8.3 versus 7.7). Participant characteristics for both timepoints are reported in detail in Supplementary Table 1.

### Uptake of a COVID-19 vaccine at T2

A large majority of participants (90.0 %; 95 % CI 88.1 %, 91.6 %, *n* = 1033/1148) reported having received two doses of a COVID-19 vaccine, with a further 2.2 % (95 % CI 1.5 %, 3.2 %; *n* = 25) reporting having had one dose; 7.4 % (95 % CI 6.0 %, 9.1 %; *n* = 85) had not been vaccinated (0.3 % [*n* = 4] preferred not to say, 0.1 % [*n* = 1] did not know).

Most participants (56.6 %, *n* = 599/1058) reported having the AstraZeneca vaccine, followed by the Pfizer-BioNTech vaccine (39.4 %, *n* = 417). Few reported having the Moderna (3.4 %, *n* = 36), another vaccine not listed (0.3 %, *n* = 3), or the Janssen (0.1 %, *n* = 1) vaccines (0.2 % did not know, *n* = 2). No one selected the made-up brand “Cambriona”. A minority (12.4 %, 95 % CI 10.5 %, 14.5 %, *n* = 131/1058) reported preferring a different vaccine to the one they had been given; 687 (64.9 %; 95 % CI 62.0 %, 67.8 %) did not prefer another vaccine; 240 (22.7 %; 95 % CI 20.3 % to 25.3 %) did not know. Of these, 90.1 % (*n* = 118/131) reported preferring to receive the Pfizer-BioNTech vaccine (5.3 % Moderna, *n* = 7; 2.3 % Janssen, *n* = 3; 1.5 % AstraZeneca, *n* = 2; 0.8 % prefer not to say, *n* = 1).

#### Factors associated with subsequently being fully vaccinated at T2

More participants had been vaccinated at T2 (October 2021) than had indicated being very likely to do so at T1 (January 2021; *n* = 1030 vaccinated, compared to *n* = 847 very likely; Table 1). Almost all participants (99.9 %) who indicated that they were very likely to be vaccinated had been vaccinated. Of those who had previously stated they were very unlikely to be vaccinated, 39.8 % had been vaccinated; 85.9 % of those who were uncertain had been vaccinated.

**Table 1:** Association between vaccination intention at T1 (January 2021, using *a priori* cut points) and subsequent vaccination status at T2 (October 2021). Data are frequencies (%).

	Vaccination intention, January 2021			
Vaccination status, October 2021	Very unlikely	Uncertain	Very likely	Total
Vaccinated	37 (39.8)	146 (85.9)	847 (99.9)	1030
Unvaccinated	56 (60.2)	24 (14.1)	1 (0.1)	81
Total	93 (100)	175 (100)	848 (100)	1111

Vaccination intention was strongly associated with vaccine uptake, with an odds ratio of 1.89 (95 % CI 1.71, 2.09) and a coefficient of discrimination of 0.443 (Table 2). Addition of the other predictors in the second block significantly improved the fit of the model (*χ*^2^ = 29.41, *df* = 7, *p* < 0.001) and raised the coefficient of discrimination to 0.501. Vaccine intention remained a significant predictor, with a slightly lower odds ratio of 1.43 (95 % CI 1.21, 1.68); however, the increase in the coefficient of discrimination of only 0.058 indicates that, after controlling for the other variables in the model, intention remained an important predictor, with the largest standardized coefficient. Three of the components – social norms relating to vaccination, necessity of vaccination, and perceived safety of the vaccine – were also significant predictors, but of less strength, as indicated by the standardized beta coefficients.

**Table 2:** Results of the logistic regression model analysing associations with vaccination intention. The odds ratios indicate the increase or decrease in the odds of vaccination for a one-unit increase in the predictor variable. The model was based on 1111 cases with complete data.

Predictor variable	Level	Odds ratio	95 % confidence interval	*p* value	Standardized beta	Coefficient of discrimination for model
**Univariable model**						
Vaccination intention	0–10	1.887	1.708, 2.085	<0.001*	0.570	0.443^†^
**Multivariable model**						
Vaccination intention	0–10	1.429	1.214, 1.681	<0.001*	0.301	0.501^#^
Component 1: social norms	—	1.639	1.164, 2.308	0.005*	0.145	
Component 2: necessity of vaccination	—	2.137	1.350, 3.383	0.001*	0.229	
Component 3: perceived safety of the vaccine	—	1.937	1.318, 2.846	0.001*	0.192	
Component 4: adequacy of information about the vaccine	—	1.233	0.908, 1.674	0.180	0.061	
Only people who are at risk of serious illness need to be vaccinated	0–10	1.073	0.944, 1.220	0.282	0.063	
Widespread vaccination is just a way to make money for vaccine manufacturers	0–10	0.937	0.825, 1.064	0.313	–0.050	
Had/will have a vaccination for influenza last/this winter	Yes	1.580	0.693, 3.600	0.277	0.067	

* *p* ≤ 0.05; † model deviance = 285.103; ^#^ model deviance = 255.689.

### Changes in beliefs and attitudes about COVID-19 illness and vaccination between January and October 2021

Compared to January 2021, in October 2021, participants perceived COVID-19 to be less severe and have a smaller impact on one’s life, but perceived their own vulnerability to COVID-19 as higher (Table 3). Participants had greater trust in COVID-19 management, perceived COVID-19 vaccination to be safer, and were more likely to perceive that they had adequate information about the vaccine, but were less likely to think that freedom from restrictions could be achieved through vaccination in October 2021. Participants were also less likely to agree that only those who are at risk of serious illness from COVID-19 need to be vaccinated and that the way that vaccines were given went against the manufacturers’ recommendation. Fear of needles also decreased between January and October 2021. Comparison of the mean change in attitudes between those who had been partially or fully vaccinated and those who had not been vaccinated indicates the extent of the difference in the rates of change between these sub-groups.

**Table 3:** Changes in attitudes to COVID-19 between T1 and T2 (differences are T2 minus T1, i.e. October 2021 minus January 2021 scores; positive differences indicate a strengthening of attitude or belief from T1 to T2, negative differences a weakening from T1 to T2). Values are given for the sample as a whole and for those participants who subsequently did or did not vaccinate (5 participants did not report vaccination status). Those already vaccinated at T1 were excluded. Interaction terms are not presented, as an attempt to calculate these produced unreliable estimates owing to the marked difference in numbers between those partially or fully vaccinated and those not vaccinated.

		**T1 Mean (SD)**	**T2 Mean (SD)**	**Mean difference (99** % **CI)**	***p* value for difference**	**Effect size, Cohen’s f**
**Beliefs and attitudes regarding COVID-19**	Component 1: perceived severity of COVID-19					
	All respondents (*n* = 1116)	4.348 (1.954)	3.442 (2.236)	–0.906 (­–1.039, –0.773)	<0.001*	–0.528
	Partially or fully vaccinated (*n* = 1030)	4.558 (1.712)	3.665 (2.048)	–0.893 (–1.028, –0.759)		–0.534
	Not vaccinated (*n* = 81)	1.828 (2.633)	0.710 (2.573)	–1.118 (–1.747, –0.489)		–0.525
	Component 2: individual vulnerability to COVID-19					
	All respondents (*n* = 1116)	4.577 (2.810)	4.981 (2.635)	0.404 (0.249, 0.559)	<0.001*	0.201
	Partially or fully vaccinated (*n* = 1030)	4.405 (2.768)	4.831 (2.600)	0.426 (0.266, 0.586)		0.215
	Not vaccinated (*n* = 81)	6.623 (2.538)	6.890 (2.400)	0.267 (–0.358, 0.892)		0.128
	Component 3: trust in COVID-19 management					
	All respondents (*n* = 1116)	6.693 (2.712)	6.894 (2.789)	0.201 (0.032, 0.370)	0.002*	0.090
	Partially or fully vaccinated (*n* = 1030)	6.824 (2.675)	7.043 (2.718)	0.219 (0.046, 0.392)		0.101
	Not vaccinated (*n* = 81)	5.191 (2.713)	5.160 (3.035)	–0.031 (–0.787, 0.725)		–0.012
	Component 4: impact of COVID-19 on one’s life					
	All respondents (*n* = 1116)	9.598 (2.366)	9.056 (2.511)	–0.542 (–0.708, –0.377)	<0.001*	–0.253
	Partially or fully vaccinated (*n* = 1030)	9.636 (2.311)	9.138 (2.429)	–0.498 (–0.667, –0.329)		–0.237
	Not vaccinated (*n* = 81)	9.082 (2.880)	8.126 (3.116)	–0.956 (–1.676, –0.235)		–0.392
**Beliefs and attitudes regarding COVID-19 vaccination**						
	Component 1: social norms					
	All respondents (*n* = 1115)	6.884 (2.973)	6.839 (3.062)	–0.046 (–0.228, 0.136)	0.519	0.019
	Partially or fully vaccinated (*n* = 1029)	7.281 (2.629)	7.322 (2.554)	0.040 (–0.146, 0.225)		0.017
	Not vaccinated (*n* = 81)	2.052 (2.616)	0.882 (2.532)	–1.170 (–1.964, –0.375)		– 0.435
	Component 2: necessity of vaccination					
	All respondents (*n* = 1115)	11.357 (2.006)	11.235 (1.976)	–0.122 (–0.289, 0.046)	0.061	–0.055
	Partially or fully vaccinated (*n* = 1029)	11.467 (1.894)	11.254 (1.837)	–0.211 (–0.377, –0.045)		–0.101
	Not vaccinated (*n* = 81)	10.169 (2.671)	11.121 (3.235)	0.952 (0.082, 1.823)		0.322
	Component 3: perceived safety of the vaccine					
	All respondents (*n* = 1115)	–9.831 (2.809)	–8.740 (2.627)	1.092 (0.894, 1.289)	<0.001*	0.427
	Partially or fully vaccinated (*n* = 1029)	–9.579 (2.648)	–8.416 (2.329)	1.157 (1.955, 1.360)		0.461
	Not vaccinated (*n* = 81)	–12.928 (2.893)	–12.682 (2.884)	0.245 (–0.638, 0.128)		0.084
	Component 4: adequacy of information about the vaccine 0.084					
	All respondents (*n* = 1115)	6.877 (2.512)	7.272 (2.367)	0.395 (0.194, 0.596)	<0.001*	0.153
	Partially or fully vaccinated (*n* = 1029)	6.852 (2.353)	7.195 (2.233)	0.344 (0.139, 0.548)		0.135
	Not vaccinated (*n* = 81)	7.088 (3.912)	8.222 (3.554)	1.133 (0.217, 2.050)		0.364
	Component 5: freedom from restrictions through the vaccine					
	All respondents (*n* = 1115)	2.109 (2.711)	1.721 (2.273)	–0.388 (–0.610, –0.167)	<0.001*	–0.135
	Partially or fully vaccinated (*n* = 1029)	2.097 (2.662)	1.709 (2.164)	–0.390 (–0.612, –0.168)		–0.143
	Not vaccinated (*n* = 81)	2.184 (3.239)	1.814 (3.389)	–0.371 (–1.543, 0.802)		–0.095
	Only people who are at risk of serious illness from coronavirus need to be vaccinated					
	All respondents (*n* = 1116)	2.270 (3.017)	1.998 (2.964)	–0.272 (–0.527, –0.016)	0.006*	–0.084
	Partially or fully vaccinated (*n* = 1030)	1.985 (2.847)	1.705 (2.761)	–0.281 (–0.548, –0.014)		–0.084
	Not vaccinated (*n* = 81)	5.827 (2.850)	5.617 (3.093)	–0.210 (–1.171, 0.751)		–0.063
	The way the coronavirus vaccines are being given goes against the manufacturers’ recommendations					
	All respondents (*n* = 1116)	4.944 (3.040)	3.111 (2.633)	–1.832 (–2.068, –1.597)	<0.001*	–0.602
	Partially or fully vaccinated (*n* = 1030)	4.868 (3.071)	2.957 (2.582)	–1.911 (–2.156, –1.665)		–0.627
	Not vaccinated (*n* = 81)	5.778 (2.470)	4.975 (2.559)	–0.802 (–1.631, 0.026)		–0.285
	Widespread coronavirus vaccination is just a way to make money for vaccine manufacturers					
	All respondents (*n* = 1116)	1.934 (2.578)	2.063 (2.680)	0.129 (–0.025, 0.283)	0.031	0.063
	Partially or fully vaccinated (*n* = 1030)	1.587 (2.176)	1.681 (2.241)	0.093 (–0.065, 0.252)		0.045
	Not vaccinated (*n* = 81)	6.099 (3.315)	6.654 (3.206)	0.556 (–0.098, 1.210)		0.250
**General vaccine beliefs and attitudes**	I am afraid of needles					
	All respondents (*n* = 1116)	2.682 (3.320)	2.488 (3.259)	–0.194 (–0.345, –0.042)	0.001*	–0.101
	Partially or fully vaccinated (*n* = 1030)	2.640 (3.298)	2.473 (3.257)	–0.167 (–0.320, –0.014)		–0.090
	Not vaccinated (*n* = 81)	3.284 (3.617)	2.605 (3.342)	–0.679 (–1.415, –0.057)		–0.272
	In general vaccination is a good thing					
	All respondents (*n* = 1116)	9.053 (1.685)	9.025 (1.719)	–0.028 (–0.121, 0.066)	0.444	0.032
	Partially or fully vaccinated (*n* = 1030)	9.302 (1.222)	9.267 (1.260)	–0.035 (–0.120, 0.050)		–0.032
	Not vaccinated (*n* = 81)	6.123 (2.960)	6.136 (3.274)	0.012 (–0.652, 0.677)		–0.005

* *p* ≤ 0.01.

### Future vaccine intentions, and reasons behind intention.

#### Receiving a second dose

Of 24 participants at T2 (October 2021) who had not completed their vaccine schedule (reported only receiving one dose), 79.2 % (95 % CI 59.5 %, 90.8 %, *n* = 19) were very likely to have a second dose; 8.3 % (95 % CI 2.3 %, 25.9 %, *n* = 2) were very unlikely, and 12.5 % (95 % CI 4.3 %, 31.0 %, *n* = 3) were uncertain (see Supplementary Fig. 1). The modal (most common) answer was the maximum value on the intention scale, with 63.5 % participants (*n* = 15) selecting “10 (extremely likely)”.

The most common reasons for having a second dose were to protect oneself, to be able to move about freely, and to protect others (Table 4). Lack of trust in authorities formed the main reasons for not being likely to have a second dose.

**Table 4:** Thematic categorization of codes generated by content analysis of reasons for or against having a second dose of the COVID-19 vaccine, by vaccination intention at T1. Data are the frequency with which codes were identified and themes are presented in descending order of overall frequency.

Theme	Codes	Vaccination intention		
		Very unlikely	Uncertain	Very likely
Protecting oneself (total = 15)	To protect oneself			10
	To follow medical advice			2
	Higher protection than single dose			2
	To have both vaccines			1
Moving about freely (total = 6)	Wanting to travel		1	3
	To gain an immunity passport			1
	Vaccine is a requirement			1
Protecting others (total = 3)	To protect the wider community			3
Lack of trust in authorities and misinformation (total = 3)	Lack of trust in government	1		
	Lack of trust in science	1		
	Conspiracy theory	1		
Safety concerns (total = 3)	Concerns about vaccine side effects		1	
	Adverse side effects with first dose	1	1	
To end pandemic (total = 2)	To overcome the pandemic			1
	Vaccine is a civic duty/social responsibility			1
Free will (total = 2)	Opposing introduction of covid passports			1
	Only want a second dose if it is a free choice			1

#### Receiving a COVID-19 vaccine

Of 90 participants who had not received any COVID-19 vaccine at T2 (October 2021), 5.6 % (95 % CI 2.4 %, 12.4 %, *n* = 5) were very likely to have a COVID-19 vaccine, 67.8 % (95 % CI 57.6 %, 77.5 %, *n* = 61) were very unlikely, and 26.7 % (95 % CI 18.6 %, 36.6 %, *n* = 24) were uncertain (see Supplementary Figure 2). The modal answer was the minimum value on the intention scale, with 53.3 % participants (*n* = 48) selecting “0 (extremely unlikely)”.

The most common reasons for not having a COVID-19 vaccine were safety concerns, perceiving the vaccine to be ineffective and preferring natural immunity (Table 5). The main reason behind intention to be vaccinated was to protect oneself.

**Table 5:** Thematic categorization of codes generated by content analysis of reasons for or against having a COVID-19 vaccine, by vaccination intention. Data show the frequencies with which codes were identified, and themes are presented in descending order of overall frequency.

Theme	Codes	Vaccination intention		
		Very unlikely	Uncertain	Very likely
Safety concerns (total = 96)	Concerns about the long-term side effects of the vaccine	17	2	
	Lack of research about the vaccine	14	5	
	Concerns about vaccine side effects	11	3	
	Vaccines are experimental using mRNA/novel technology	8		
	Concerns about the quick development of the vaccine	5	1	
	Having heard negative stories about vaccine	3		
	Vaccines are riskier than virus	3		
	Knowing of people who had adverse effects	3	1	
	Concerns about vaccine safety	2		
	Pregnancy concerns	2		1
	Interference of the vaccine with other health conditions	2	1	
	Vaccine more harmful than the virus	1		
	Does not want to be a guinea pig	1		
	Allergy concerns	1		
	Fear of developing myocarditis	1		
	Suffers with anxiety	1		
	Concerns about vaccine composition	1		
	Adverse effects from previous vaccines	1		
	Concerns about setting precedents about bodily autonomy		1	
	Fertility concerns		1	
	Vaccine can interfere with menstrual cycle		1	
Vaccine not effective (total = 24)	Vaccine does not stop covid transmission	10	1	
	Concerns about the effectiveness of vaccine	6		
	Lack of trust in the vaccine	4	1	
	Concerns around the need for repeated booster shots	1	1	
	Doubt about the effectiveness of the vaccine against different variants	1		
	Other preventative measures are more effective		1	
Natural immunity (total = 21)	Natural immunity is sufficient	7	3	
	Higher perceived immunity from catching the virus	3		
	Likely or already had the virus	5	1	
	Prefer natural treatments	1	1	
Negative vaccine views (total = 9)	Vaccine is unnecessary	5		
	Does not have flu jab	2		
	Prefers not to have the vaccine	1		
	Anti-vaccine in general	1		
For protection (total = 9)	To protect oneself		1	3
	To protect the wider community			1
	Perceived high personal risk of disease severity		1	
	Anxiety about the virus		1	
	Vaccine reduces disease severity/fatality		1	
	Trust in science			1
Sources of influence (total = 8)	Influenced by the media	3	1	
	Put off by societal pressure	1	2	
	Social influence		1	
Lack of trust in authorities (total = 7)	Loss of trust due to changing government guidelines	1		
	Lack of trust in science	1	1	
	Lack of trust in media transparency	1	1	
	Vaccine too politicized		1	
	Lack of trust in government		1	
No personal need (total = 7)	No personal need for the vaccine	2	2	
	Only high-risk need the vaccine	1		
	Enough people have been vaccinated	1		
	Would have vaccine if high risk		1	
Misinformation (total = 7)	Conspiracy theory	4	1	
	Conflicting information	1		
	Vaccine creates a new strand	1		
Low threat appraisal (total = 5)	Perceived low personal risk of disease severity	3		
	High survival rate of the virus	2		
Future intention (total = 5)	May get the vaccine in the future		2	
	Uncertain		1	
	Planning on getting vaccinated eventually		1	
	If vaccine becomes a requirement		1	
Overreaction (total = 3)	Too much fuss is being made about the virus	2		
	Tendency to “overvaccinate”		1	
Perceived knowledge insufficiency (total = 2)	Lack of knowledge about the vaccine	1		
	Lack of knowledge about the virus and variants	1		
Access problem (total = 2)	No vaccine centre nearby		1	
	Unable to have vaccine right now		1	
Move about freely (total = 2)	Wanting to travel		1	1

#### Booster vaccination

**Fig. 1:**
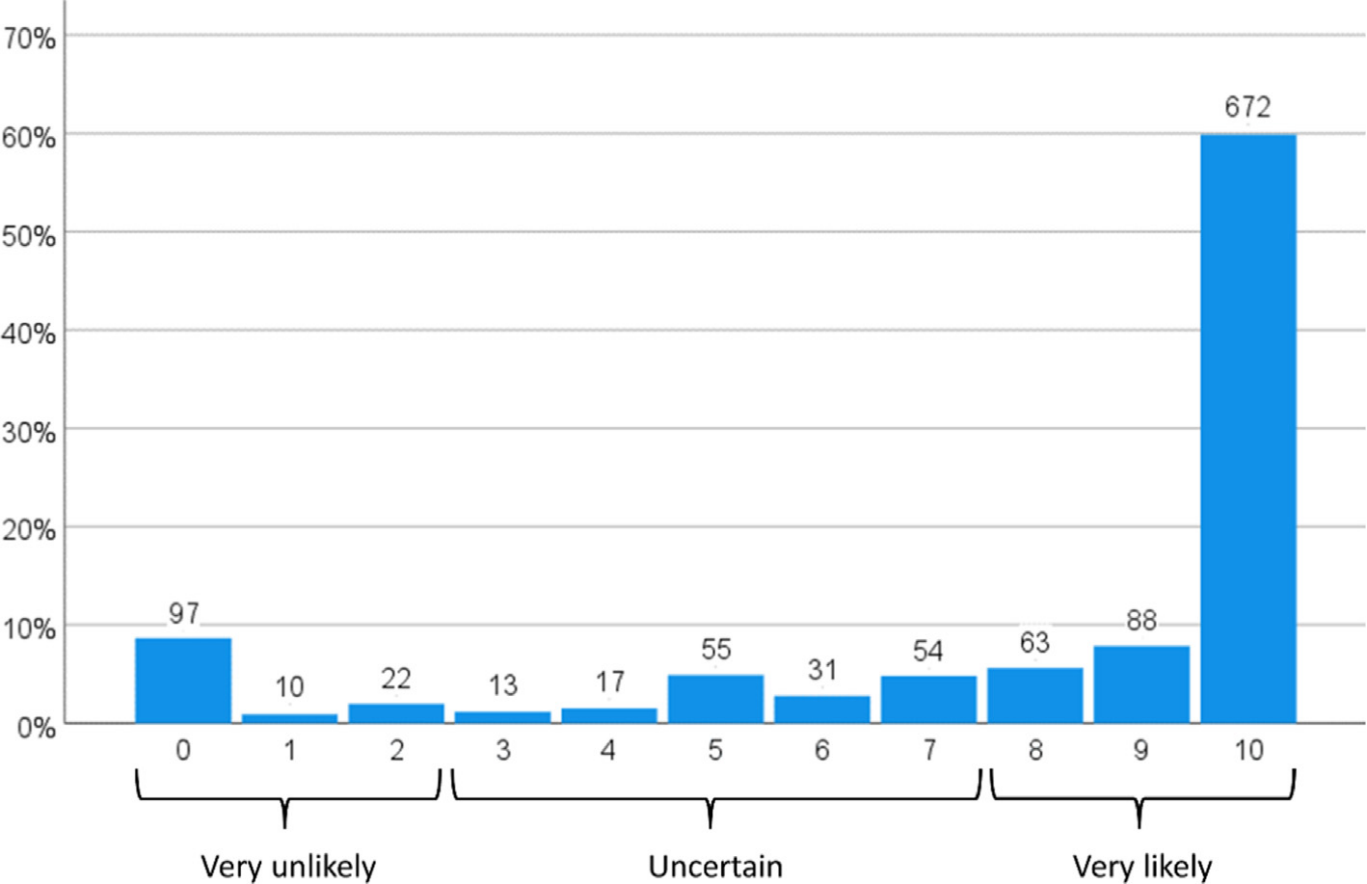
Likelihood of having a COVID-19 booster vaccine, on a scale labelled ‘extremely unlikely’ (0) to 10 ‘extremely likely’ (10), with *a priori* cut-points used to categorize respondents in terms of their booster vaccination intention (*n* = 1122).

Twenty-five participants (2.2 %) had already received a COVID-19 booster; these people were excluded from further questions about booster vaccination. Of the remaining 1122 participants, 73.4 % (95 % CI 70.8 %, 75.9 %, *n* = 823) reported being very likely to receive a COVID-19 booster vaccine if one became available to them; 11.5 % (95 % CI 9.6 % to 13.4 %, *n* = 129) were very unlikely to do so and 15.2 % (95 % CI 13.1 %, 17.3 %, *n* = 170) were uncertain. The modal answer was the maximum value on the intention scale, with 59.8 % participants (*n* = 672) selecting “10 (extremely likely)” (Fig. 1).

## Discussion

In our sample, 90 % of participants reported having received two COVID-19 vaccines, with a further 2 % reporting having had one dose. Vaccination uptake was associated with higher vaccination intention, greater perceived social norms for, necessity of, and safety of, vaccination. Of participants who had had only one vaccine dose, most indicated that they were likely to have a second dose. The most common reasons for this were to protect oneself and others, and to be able to move about freely. Of participants who had not had a COVID-19 vaccine, most reported being unlikely to have one, with the most common reasons being safety concerns, perceiving the vaccine to be ineffective and preferring natural immunity.

More people reported having been vaccinated than had previously reported intending to be vaccinated [1]. This is unusual, as intentions for health behaviours are generally higher than subsequently enacted behaviours [33]. This is good news for the vaccination campaign in the UK. Our results are not directly comparable to official vaccine statistics, as official figures report on vaccine uptake in those aged 16 years or over (our sample was limited to those aged 18 years and older) [50]. Data stratified by age are available only for England [51]. Most of our participants reported receiving the AstraZeneca vaccine (57 %). Among participants who would have preferred to receive a different vaccine, the overwhelming majority (90 %) would rather have received the Pfizer-BioNTech vaccine. This is likely due to the widely publicized associations between adverse effects and the AstraZeneca vaccine [14].

Vaccine uptake was strongly associated with previous vaccination intention in our study. An increase of one point on the 0–10 intention scale was associated with approximately a 43 % increase in the odds of vaccination, accounting for other variables that we analysed in the regression model. This association is in line with theoretical models of health behaviour (e.g. Protection Motivation Theory [52], and COM-B framework of behaviour [53]), and previous research conducted in other countries [35], [36], [37]. Other psychological factors were also associated with vaccine uptake, namely greater perceived social norms relating to vaccination, greater perceived necessity for vaccination, and greater perceived safety of the vaccine. These factors have also been associated with COVID-19 vaccination intention in other UK studies [1], [2], [4]. The COM-B framework of behaviour states that capability, opportunity and motivation must be present in order for a behaviour to occur [53]. Vaccination intention, perceived necessity and perceived safety all fall under motivation – the mental processes that influence decision making. Perceived social norms are categorized under opportunity (external factors that facilitate or allow a behaviour). However, capability (the ability to carry out the behaviour) is also important, and encompasses, for example, access to vaccination clinics. In England, people aged 50 years and older, residents in care homes, health and social care staff, and those in a clinical risk group (and who are aged 5 years and older) will be offered a COVID-19 vaccination in Autumn 2022 [54]. Further research is needed to see whether restricted eligibility criteria (compared to the entire population) and less widespread vaccine clinics affect uptake in these groups, and whether psychosocial factors are more influential in the vaccination decision.

Of those who had received one dose of the vaccine, intention to have a second dose was high, with the main reason being to protect oneself. In a sample of UK respondents who had not yet decided whether to be vaccinated or who thought they would probably not be vaccinated (conducted October 2020), intention to receive a COVID-19 vaccine was associated with more positive vaccine attitudes and greater perceived safety of vaccination, among other factors [55]. The pattern in participants who had not received any COVID-19 vaccine was different, with most not intending to be vaccinated in future. The main reasons for this were related to safety concerns surrounding the vaccine, perceptions that the vaccine was not effective, and preferring natural immunity. These factors were also associated with vaccine refusal in previous pandemics [56]. Taken together, these results suggest that, at a stage where all UK adults had been offered vaccination, those who had started the vaccine programme were likely to complete it, while those who had not received any vaccine were unlikely to do so in the future. Communications should emphasize the safety, effectiveness, and mostly mild side-effects of the COVID-19 vaccine to further increase early uptake. Research shows that it may be difficult to change people’s attitudes and beliefs regarding vaccination, and where change does occur, there may not be a great effect on vaccine uptake [57]. However, a recent systematic review investigating the effectiveness of interventions at increasing COVID-19 vaccine uptake has found that messages that communicate the safety and effectiveness of the vaccine increased uptake [58].

There was evidence for changes in beliefs and attitudes about COVID-19, COVID-19 vaccination, and general vaccine beliefs between January and October 2021. Participants perceived themselves as being more susceptible to COVID-19, but perceived the illness as less severe. This is likely to reflect the predominant strain circulating in the UK at both timepoints (January 2021: alpha, October 2021: delta). How these changes in beliefs and attitudes affect uptake of booster vaccination remains to be seen. A previous systematic review of vaccine uptake indicated that there was strong evidence that vaccination was associated with perceived susceptibility to infection, but weak evidence for an association between perceived severity of infection; likely because one may consider the likelihood of catching the illness before evaluating its severity [59]. In October 2021, participants perceived COVID-19 as having a smaller impact on one’s life and there was less emphasis on freedom of restrictions through the vaccine. This may reflect the removal of legal restrictions on mixing in England on 19 July 2021. In contrast to a study of older adults in the UK, which found that worries about unforeseen future effects had increased (between May 2020 and May 2021) [43], we found that perceptions of COVID-19 vaccine safety increased between January and October 2021 in those who were vaccinated (no change in those who were not vaccinated). Data collection for the study of older adults was carried out one month after safety concerns about the AstraZeneca vaccine (given to most of the UK population aged 40 years and above) were published in the media [14]. Contrary to a study conducted in Italy [42], we found no evidence for a change in beliefs about commercial profiteering. There were some cases where changes in attitudes and beliefs differed by vaccination status. For example, perceived social norms for vaccination decreased in those not vaccinated, but stayed stable in those who were vaccinated. This may be due to participants searching for, or being shown, information that confirms their own beliefs, resulting in “echo chambers” of one’s own beliefs [60].

Most participants in our study (73.4 %) intended to receive a booster vaccine when one became available to them. This is lower than in another UK study (conducted November to December 2021), which found that 8 % of participants were unwilling to receive or uncertain about receiving a COVID-19 booster [61]. This difference may be explained by the fact that their sample comprised only fully vaccinated people. Factors associated with not intending to receive a COVID-19 booster included low levels of stress about catching or becoming seriously ill with COVID-19 [61]. How recurring (likely yearly) COVID-19 vaccination campaigns affect vaccine intention and uptake remains to be seen.

One strength of this study is its longitudinal nature, with participants completing one survey at the start of the COVID-19 vaccine rollout in the UK and another when two doses of the vaccine had been offered to all UK adults. People who completed our T2 survey had higher vaccination intentions than those who did not. Few people indicated that they had received no or just one vaccine dose at T2. Data are self-reported and so are potentially subject to social desirability bias. However, the anonymous nature of the survey should mitigate this. We did not investigate capability factors, e.g. access to vaccine clinics, in logistic regression analyses. Nor was it mentioned spontaneously by participants in open-text responses included in content analyses. In 2021, rollout of COVID-19 vaccines in the UK was widespread. Capability issues may be more likely to arise at times when access to vaccination is less extensive.

Official figures show that uptake of the COVID-19 vaccine has been high; this is reflected in self-reported uptake in our sample. Vaccine uptake was associated with higher vaccination intention, perceived safety of vaccination, perceived necessity of vaccination, and social norms for vaccination. Where participants had initiated the vaccine programme, they indicated being likely to complete the vaccination schedule. Where participants had not received any COVID-19 vaccination, they reported being unlikely to begin it. Communications highlighting that severe adverse effects from vaccination are rare and that vaccines are effective may help increase uptake in this group.

## Author contributions

LS, JS, RA, NS, GJR and SMS conceptualized and acquired funding for the study. SMS programmed the survey, curated the data and was responsible for the administration of the project. LS, JS, MC and HD undertook formal analyses. LS wrote the original draft of the manuscript, with support from JS for details of statistical analyses and results. MC, HD, RA, NS, GJR and SMS reviewed and edited drafts.

## Source of funding

Data collection was funded by a Keele University Faculty of Natural Sciences Research Development award to SMS, JS and NS, and a King's COVID Appeal Fund award granted jointly to LS, GJR, RA, NS, SMS and JS. LS, RA and GJR are supported by the National Institute for Health Research Health Protection Research Unit (NIHR HPRU) in Emergency Preparedness and Response, a partnership between the UK Health Security Agency, King’s College London and the University of East Anglia. NS’s research is supported by the National Institute for Health Research (NIHR) Applied Research Collaboration (ARC) South London at King’s College Hospital NHS Foundation Trust. NS is a member of King’s Improvement Science, which offers co-funding to the NIHR ARC South London and is funded by King’s Health Partners (Guy’s and St Thomas’ NHS Foundation Trust, King’s College Hospital NHS Foundation Trust, King’s College London and South London and Maudsley NHS Foundation Trust), and the Guy’s and St Thomas’ Foundation. The views expressed are those of the authors and not necessarily those of the NIHR, the charities, UK Health Security Agency or the Department of Health and Social Care.

## Data sharing

Data are available online [45].

## Declaration of Competing Interest

The authors declare the following financial interests/personal relationships which may be considered as potential competing interests: NS is the director of the London Safety and Training Solutions Ltd, which offers training in patient safety, implementation solutions and human factors to healthcare organizations and the pharmaceutical industry. At the time of writing GJR is acting as an expert witness in an unrelated case involving a life sciences company, supported by LS. The other authors have no conflicts of interest to declare. LS, RA and GJR were participants of the Scientific Advisory Group for Emergencies or its subgroups.

## Data Availability

Data are available online at https://osf.io/tehg8/.
