# Supplementary material for: Psychosocial factors affecting COVID-19 vaccine uptake in the UK: A prospective cohort study (CoVAccS – Wave 3)
Source: Vaccine X. 2023 Feb 13;13:100276. doi: 10.1016/j.jvacx.2023.100276 (PMC9924044; doi:10.1016/j.jvacx.2023.100276)
Supplement: Supplementary data 1 [file mmc1.docx]

# Supplementary materials

**Supplementary Table 1.** Participant characteristics.^[[1]](#footnote-1)^

|  |  | TI [total n=1500] | T2 [total n=1148] |
| --- | --- | --- | --- |
| Personal and clinical characteristics | Level | *n* (%) | *n* (%) |
| Sex | Male | 728 (48.5) | 534 (46.5) |
|  | Female | 765 (51.0) | 611 (53.2) |
|  | Other | 6 (0.1) | 2 (0.2) |
|  | Prefer not to say | 1 (0.1) | 1 (0.1) |
| Age (at T1) | In years, mean (SD) | 45.6 (15.6) | 48.2 (15.1) |
| Ethnicity | White | 1269 (84.6) | 988 (86.1) |
|  | Black and minority ethnic | 224 (14.9) | 154 (13.4) |
|  | Prefer not to say | 7 (0.5) | 6 (0.5) |
| Religion | No religion | 793 (52.9) | 604 (52.6) |
|  | Christian | 571 (38.1) | 453 (39.5) |
|  | Other religion | 114 (7.5) | 76 (6.6) |
|  | Prefer not to say | 22 (1.5) | 15 (1.3) |
| Highest qualification | Degree equivalent or higher^+^ | 817 (54.5) | 621 (54.1) |
|  | Other or no qualifications | 677 (45.1) | 520 (45.3) |
|  | Prefer not to say | 6 (0.4) | 5 (0.4) |
| Employment status | Full-time | 649 (43.3) | 479 (41.7) |
|  | Part-time | 269 (17.9) | 209 (18.2) |
|  | Not working/other | 572 (38.1) | 454 (39.5) |
|  | Don’t know | 1 (0.1) | 1 (0.1) |
|  | Prefer not to say | 9 (0.6) | 5 (0.4) |
| Total household income | Under £10,000 | 94 (6.3) | 68 (5.9) |
|  | £10,000–£19,999 | 215 (14.3) | 167 (14.5) |
|  | £20,000–£29,999 | 249 (16.6) | 196 (17.1) |
|  | £30,000–£39,999 | 236 (15.7) | 182 (15.9) |
|  | £40,000–£49,999 | 179 (11.9) | 137 (11.9) |
|  | £50,000–£74,999 | 261 (17.4) | 199 (17.3) |
|  | £75,000 or over | 161 (10.7) | 117 (10.2) |
|  | Don’t know | 18 (1.2) | 8 (0.7) |
|  | Prefer not to say | 87 (5.8) | 74 (6.4) |
| Region where respondent lives | East Midlands | 127 (8.5) | 93 (8.1) |
|  | East of England | 111 (7.4) | 77 (6.7) |
|  | London | 205 (13.7) | 154 (13.4) |
|  | North East | 61 (4.1) | 50 (4.4) |
|  | North West | 176 (11.7) | 142 (12.4) |
|  | Northern Ireland | 27 (1.8) | 19 (1.7) |
|  | Scotland | 116 (7.7) | 94 (8.2) |
|  | South East | 239 (15.9) | 183 (15.9) |
|  | South West | 131 (8.7) | 102 (8.9) |
|  | Wales | 56 (3.7) | 42 (3.7) |
|  | West Midlands | 122 (8.1) | 92 (8.0) |
|  | Yorkshire and the Humber | 127 (8.5) | 99 (8.6) |
|  | Prefer not to say | 2 (0.1) | 1 (0.1) |
| Number of people in household | 1 | 233 (15.5) | 190 (16.6) |
|  | 2† | 587 (39.1) | 469 (40.9) |
|  | 3–4 | 563 (37.5) | 417 (36.3) |
|  | 5–6 | 105 (7.0) | 66 (5.7) |
|  | 7 or more | 9 (0.6) | 4 (0.3) |
|  | Prefer not to say | 3 (0.2) | 2 (0.2) |
| Extremely clinically vulnerable – respondent | Yes | 344 (22.9) | 250 (21.8) |
|  | No/prefer not to say | 1156 (77.1) | 898 (78.2) |
| Extremely clinically vulnerable – other(s) in household | Yes | 254 (16.9) | 217 (18.9) |
|  | No/not applicable/prefer not to say | 1246 (83.1) | 931 (81.1) |
| Influenza vaccination last winter (measured at T1: September 2019 to January 2020) | Yes | 457 (30.5) | 377 (32.8) |
|  | No | 1040 (69.3) | 771 (67.2) |
|  | Don’t know | 1 (0.1) | 0 (0.0) |
|  | Prefer not to say | 2 (0.1) | 0 (0.0) |
| Influenza vaccine this winter (measured at T1: September 2020 onwards) | Yes | 581 (38.7) | 494 (43.0) |
|  | No, but intend to | 180 (12.0) | 124 (10.8) |
|  | No, and don’t intend to | 723 (48.2) | 521 (45.4) |
|  | Don’t know | 13 (0.9) | 9 (0.8) |
|  | Prefer not to say | 3 (0.2) | 0 (0.0) |
| Influenza vaccination last winter (measured at T2: September 2020 to January 2021) | Yes | - | 512 (44.6) |
|  | No | - | 631 (55.0) |
|  | Don’t know | - | 2 (0.2) |
|  | Prefer not to say | - | 3 (0.3) |
| Influenza vaccine this winter (measured at T2: September 2021 onwards) | Yes | - | 208 (18.1) |
|  | No | - | 933 (81.3) |
|  | Don’t know | - | 4 (0.3) |
|  | Prefer not to say | - | 3 (0.3) |
| COVID-19 vaccination intention (measured at T1) | 0 = extremely unlikely to 10 = extremely likely, mean (SD) | 8.1 (3.0) | 8.3 (2.9) |

+ Undergraduate (e.g. BA, BSc) or postgraduate (e.g. MA, MSc, PhD) degree or other technical, professional or higher qualification.


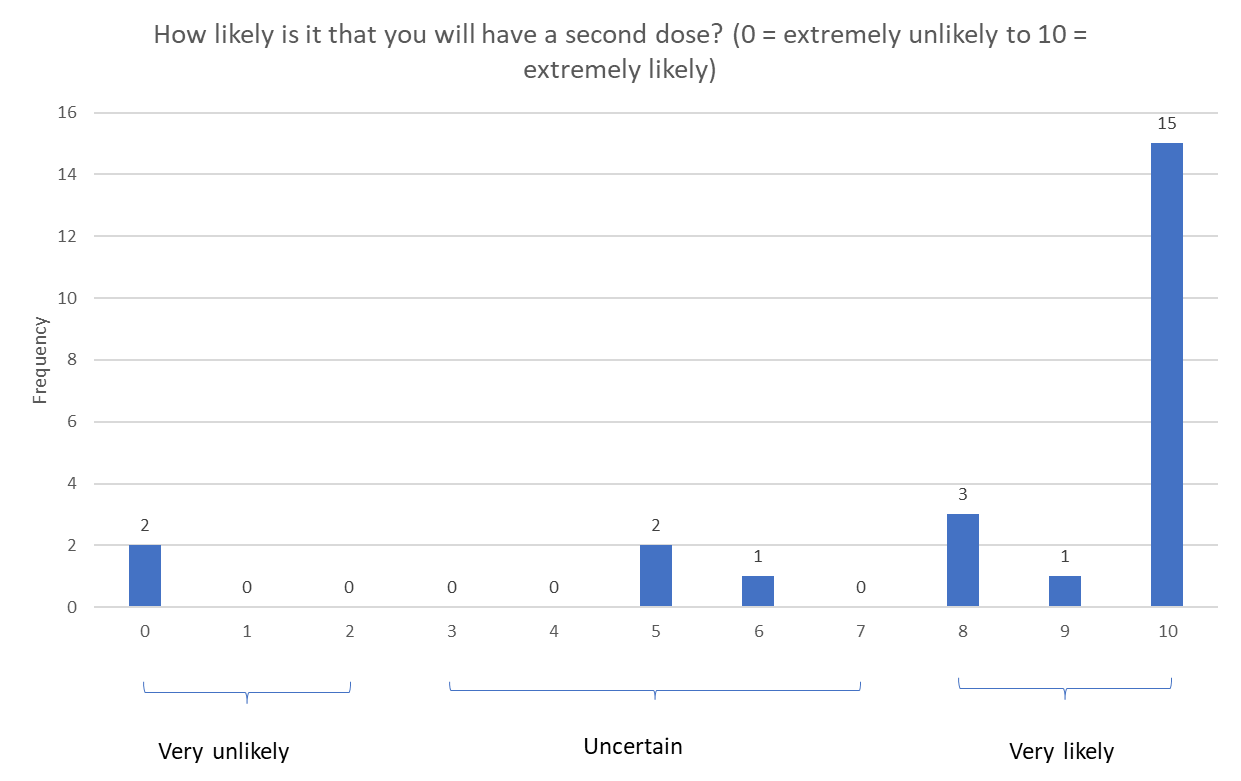


Supplementary Figure 1. Perceived likelihood of having a second vaccine dose, with *a-priori* cut-points used to categorize respondents in terms of their vaccination intention.


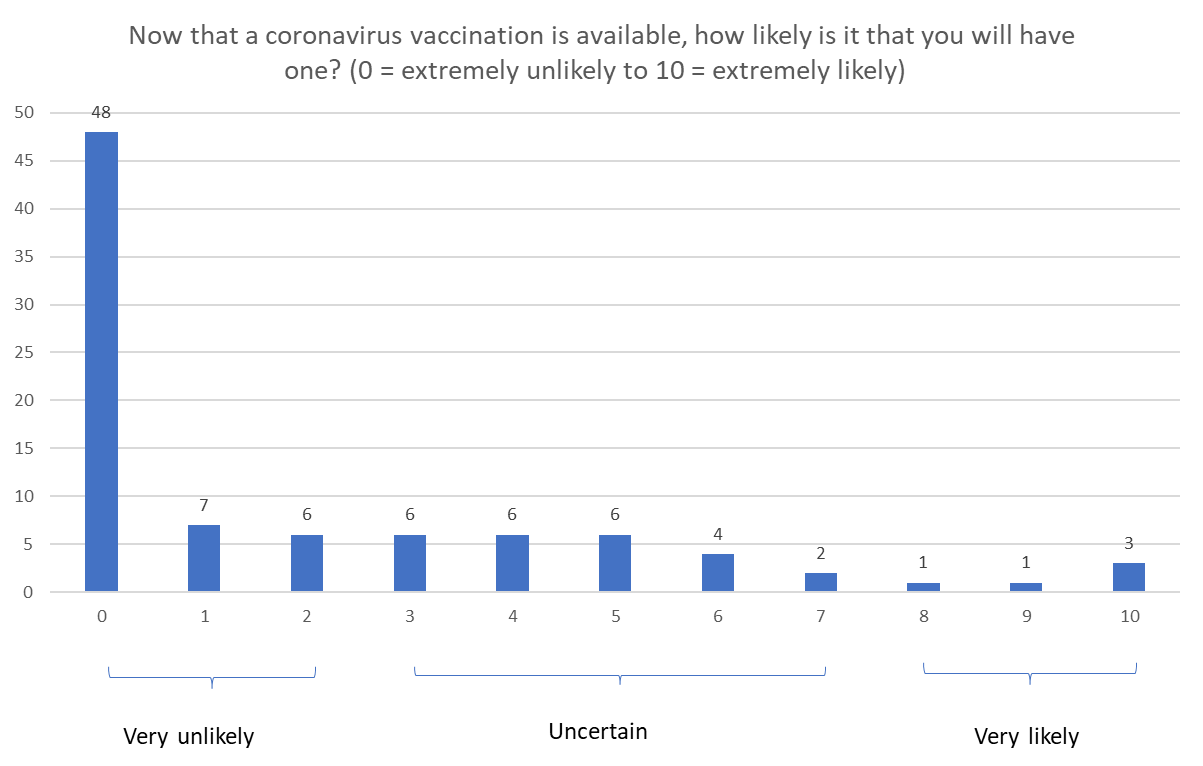

Supplementary Figure 2. Perceived likelihood of having a COVID-19 vaccine, with *a-priori* cut-points used to categorize respondents in terms of their vaccination intention (*n*=90).

1. Participant characteristics at T1 have previously been reported in Sherman et al. 2022,^1^ but are reported here for clarity and completeness. [↑](#footnote-ref-1)
